# Proliferative Myositis in the Upper Extremity: Vaccination-Induced Pathology?

**DOI:** 10.5334/jbsr.3316

**Published:** 2023-11-03

**Authors:** Tom Van der Stricht, Michaela Kubincová, Yannick De Brucker

**Affiliations:** 1 Department of Radiology, UZ Brussel, VUB, Brussels, Belgium; 2 Department of Radiology, Radiologie Buggenhout, Buggenhout, Belgium

**Keywords:** Ultrasound, MRI, Proliferative Myositis, Vaccination

## Abstract

Proliferative myositis (PM) is a rare type of inflammatory myositis. It is a benign and self-limiting pathology, but its clinical presentation can simulate malignancy. This pictorial essay illustrates the typical imaging findings of PM on ultrasound (US) and magnetic resonance imaging (MRI) in three patients. Clinical history in all three patients revealed recent vaccination procedures.

## Introduction

PM is a rare type of inflammatory myositis that presents as a rapidly growing, solid, and possibly painful mass. It is mostly seen in middle-aged people. The muscles of the trunk, upper extremities, head, and neck are most often affected [1,2].

The precise etiology remains unclear. Local trauma has been suggested as a possible cause [2]. To our knowledge, recent vaccination as a possible cause has not yet been described in the literature.

PM has typical imaging findings on ultrasound (US) and magnetic resonance imaging (MRI). It is a benign, self-limiting pathology; hence no specific therapy is needed. A wait-and-see approach with follow-up imaging is recommended, but a biopsy is often performed to rule out malignancy [1,3].

## Cases

We encountered three patients who presented with a rapidly growing mass in a muscle of the upper extremity. All our patients were vaccinated about four weeks before the onset of symptoms. Laboratory findings were normal, and there was no history of trauma. A summary of each patient’s history can be found in Table 1.

**Table 1:** Patient History.


PATIENT	AGE (y)	SEX	AFFECTED MUSCLE	VACCINATION

1	74	Male	Right brachialis muscle	Influenza

2	66	Male	Left brachioradialis muscle	COVID-19

3	64	Male	Right deltoid muscle	COVID-19


**Figure 1:**
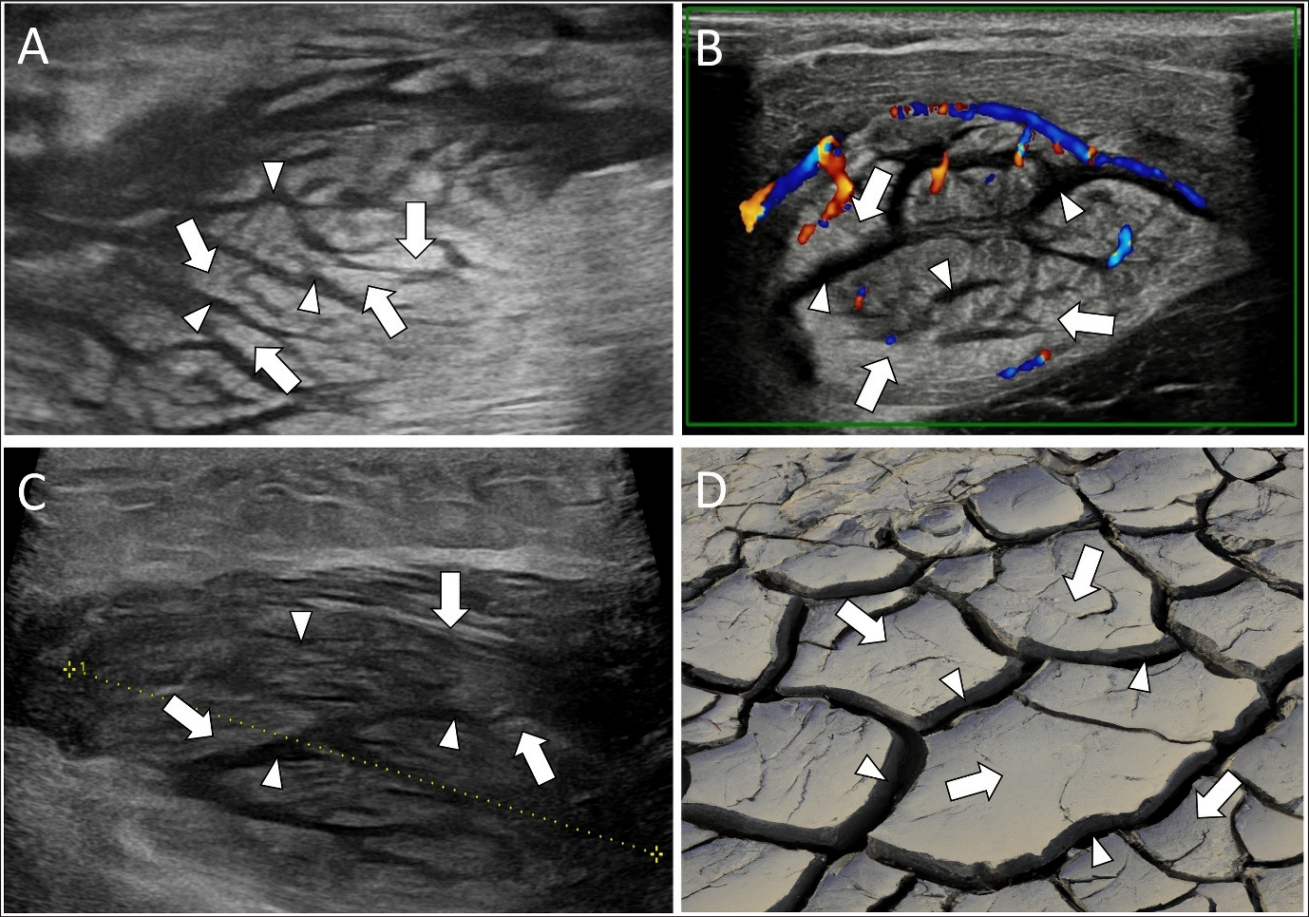
US showing a ‘cracked dry mud’ pattern of PM with swollen hyperechoic muscle fibers (white arrows) interspersed with hypoechoic bands (white arrowheads). **A:** Transverse image of the right brachialis muscle of patient 1. **B:** Transverse color Doppler image of the left brachioradialis muscle of patient 2 showing hypervascularity. **C:** Longitudinal image of the right deltoid muscle of patient 3. **D:** ‘Cracked dry mud’.

US revealed a focally swollen muscle with intact but hyperechoic muscle fibers interspersed with hypoechoic bands, giving a ‘cracked dry mud’ appearance (Figure 1) [2].

**Figure 2:**
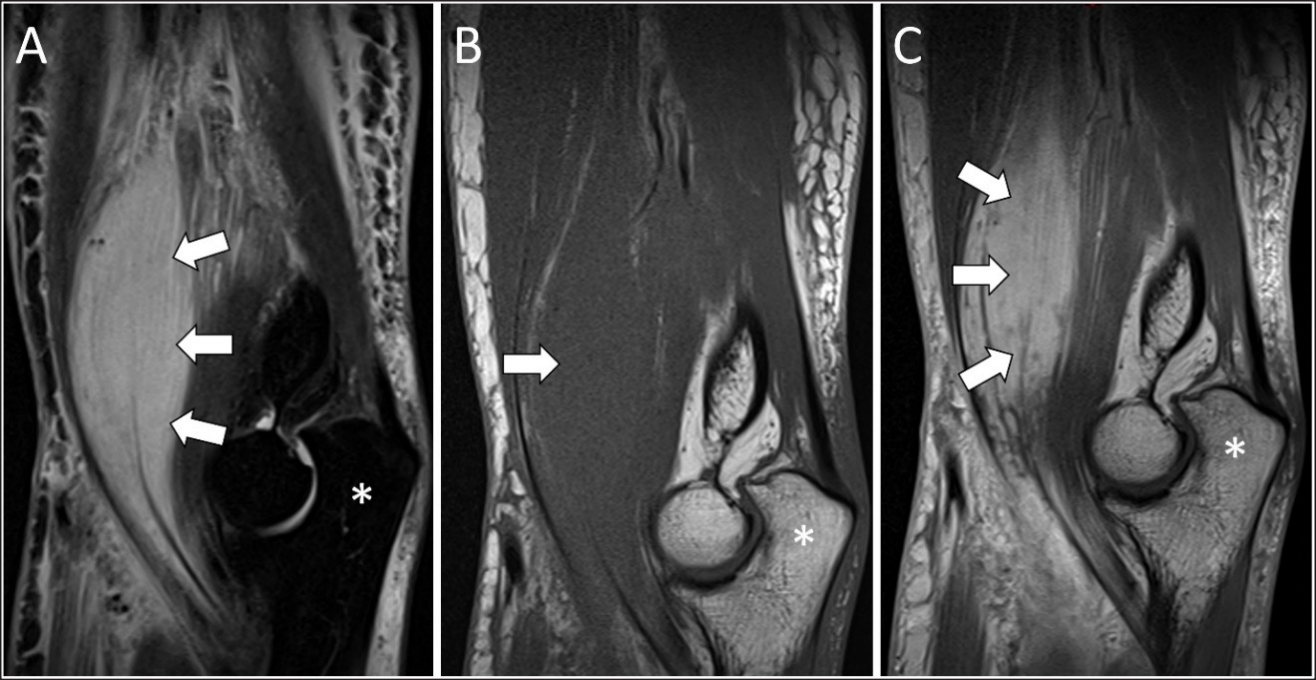
Sagittal MRI images of PM (white arrows) in the right brachialis muscle: hyperintense on STIR images **(A)**, isointense compared to the muscle on T1WI **(B)**, and a marked enhancement on post-contrast T1WI **(C)**. Olecranon (*).

**Figure 3:**
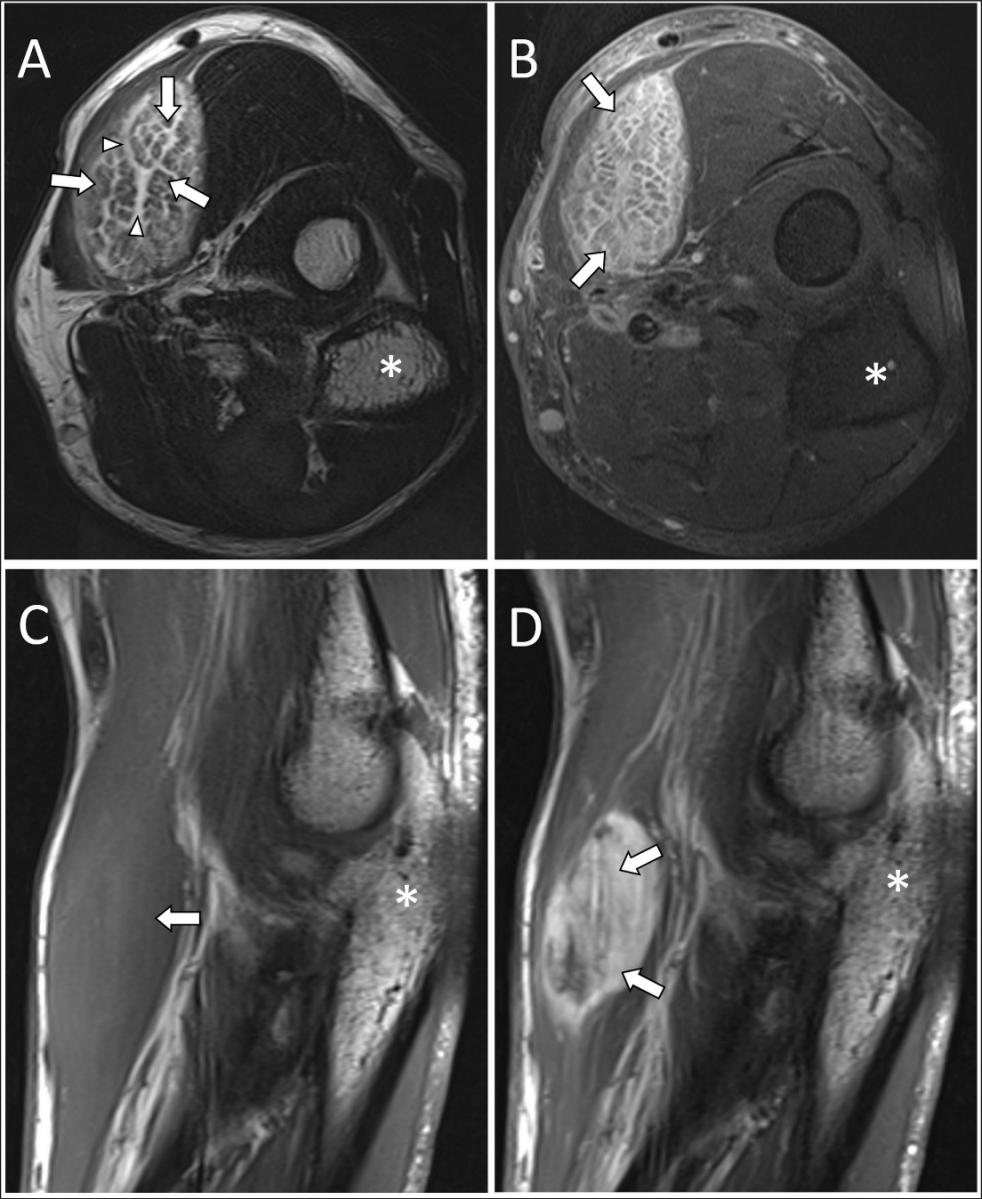
MRI images of PM in the left brachioradialis muscle. Axial T2WI **(A)**, axial post-contrast T1WI fat-saturated **(B)**, sagittal T1WI **(C)**, and sagittal post-contrast T1WI **(D)** images demonstrate a T2 hyperintense lesion with a marked enhancement. The T2WI **(A)** shows hyperintense bands (white arrowheads) interspersed with intact muscle fibers (white arrows). Olecranon (*).

**Figure 4:**
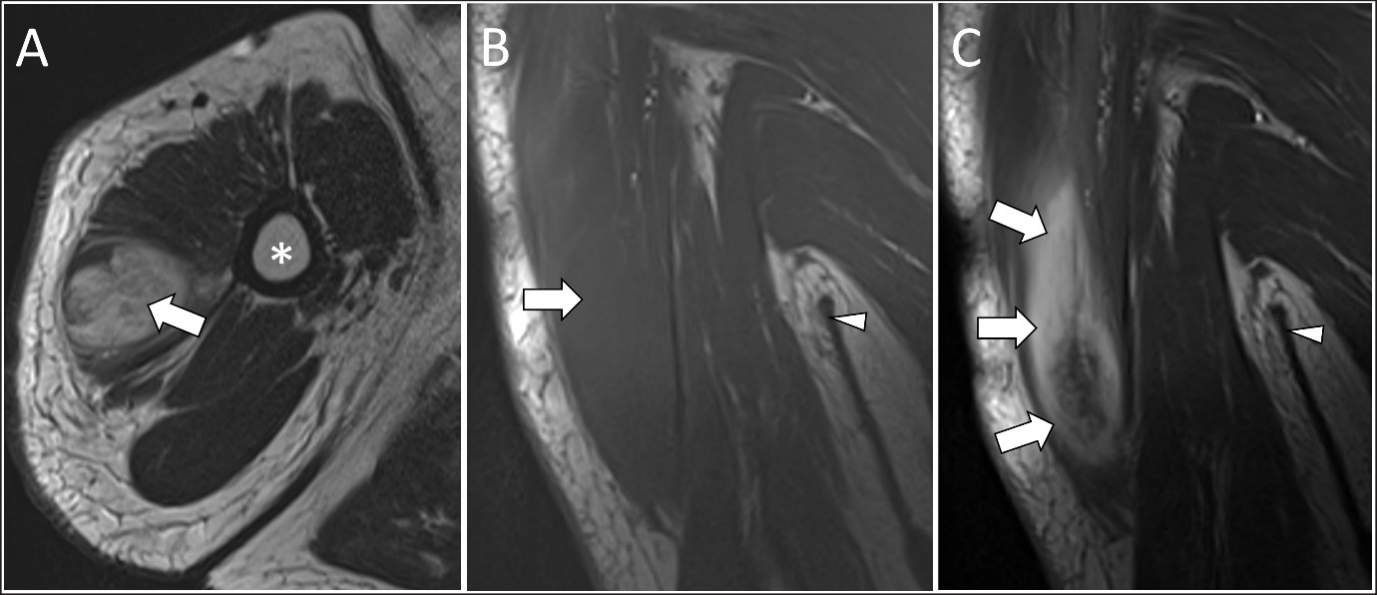
MRI images of PM in the right deltoid muscle demonstrate a hyperintense lesion (white arrow) on axial T2WI **(A)** and an isointense lesion on coronal T1WI **(B)** with a marked enhancement on coronal post-contrast T1WI **(C)** (white arrows). Humerus (*). Axilla (white arrowhead).

Subsequent MRI showed an isointense mass compared to the normal muscle on T1-weighted images (T1WI). Short tau inversion recovery (STIR) and T2-weighted images (T2WI) demonstrated a hyperintense mass with intact hypointense muscle fibers (Figures 2, 3, 4). Post-contrast T1WI showed a marked enhancement (Figures 2, 3, 4). The ‘cracked dry mud’ pattern seen on US is also demonstrated on axial fluid-sensitive sequences (Figures 3, 4) [1,2].

The diagnosis of PM was confirmed with a biopsy in the first patient. In the other two patients, a wait-and-see approach was chosen. Follow-up US revealed complete remission in all patients.

## Discussion

PM can be suspicious of a malignant lesion because of its rapid growth. Recognizing this benign pathology on imaging is important to avoid unnecessary biopsy or surgery.

US is usually the first imaging modality of choice. It can show the ‘cracked dry mud’ pattern in which the hypoechoic bands represent inflammatory infiltration [2]. MRI is often done for further evaluation. Axial fluid-sensitive sequences show a similar pattern as seen in ultrasound in which the hyperintense bands represent inflammatory infiltration [1,2].

Recognizing the intact muscle fibers is essential for making the correct diagnosis [1].

Our patients developed PM approximately four weeks after vaccination in the affected upper arm. To our knowledge, recent vaccination as a possible cause of PM has not been described in the literature.

Different subtypes of inflammatory myopathy have been described by Ding et al. in a systematic review as a side effect of COVID-19 vaccination. Jedidi et al. revealed that there is an increased risk of developing myopathy within six weeks following influenza vaccination. However, they did not describe PM as a possible side effect [4,5].

## Conclusion

Proliferative myositis has typical imaging features on US and MRI. Because PM is a benign, self-limiting pathology, a wait-and-see approach with follow-up imaging is justified. Our study does not allow us to statistically determine that vaccination is associated with a risk of developing PM, but this case series suggests that there is a possible association that may warrant further investigation.
